# Benthic Diatom Communities in an Alpine River Impacted by Waste Water Treatment Effluents as Revealed Using DNA Metabarcoding

**DOI:** 10.3389/fmicb.2019.00653

**Published:** 2019-04-09

**Authors:** Teofana Chonova, Rainer Kurmayer, Frédéric Rimet, Jérôme Labanowski, Valentin Vasselon, François Keck, Paul Illmer, Agnès Bouchez

**Affiliations:** ^1^Research Department for Limnology, Mondsee, Faculty of Biology, University of Innsbruck, Mondsee, Austria; ^2^UMR CARRTEL, INRA, Université Savoie Mont Blanc, Thonon-les-Bains, France; ^3^UMR IC2MP 7285, CNRS, Université de Poitiers, ENSIP, Poitiers, France; ^4^Department of Aquatic Sciences and Assessment, Faculty of Natural Resources and Agricultural Sciences, Swedish University of Agricultural Sciences, Uppsala, Sweden; ^5^Department of Microbiology, Faculty of Biology, University of Innsbruck, Innsbruck, Austria

**Keywords:** pharmaceuticals, diatom communities, WWTP effluents, DNA metabarcoding, functional traits, water quality index, indicator species analysis

## Abstract

Freshwater ecosystems are continuously affected by anthropogenic pressure. One of the main sources of contamination comes from wastewater treatment plant (WWTP) effluents that contain wide range of micro- and macropollutants. Chemical composition, toxicity levels and impact of treated effluents (TEs) on the recipient aquatic ecosystems may strongly differ depending on the wastewater origin. Compared to urban TEs, hospital ones may contain more active pharmaceutical substances. Benthic diatoms are relevant ecological indicators because of their high species and ecological diversity and rapid response to human pressure. They are routinely used for water quality monitoring. However, there is a knowledge gap on diatom communities’ development and behavior in treated wastewater in relation to prevailing micro- and macropollutants. In this study, we aim to (1) investigate the response of diatom communities to urban and hospital TEs, and (2) evaluate TEs effect on communities in the recipient river. Environmental biofilms were colonized in TEs and the recipient river up- and downstream from the WWTP output to study benthic diatoms using DNA metabarcoding combined with high-throughput sequencing (HTS). In parallel, concentrations of nutrients, pharmaceuticals and seasonal conditions were recorded. Diatom metabarcoding showed that benthic communities differed strongly in their diversity and structure depending on the habitat. TE sites were generally dominated by few genera with polysaprobic preferences belonging to the motile guild, while river sites favored diverse communities from oligotrophic and oligosaprobic groups. Seasonal changes were visible to lower extent. To categorize parameters important for diatom changes we performed redundancy analysis which suggested that communities within TE sites were associated to higher concentrations of beta-blockers and non-steroidal anti-inflammatory drugs in urban effluents vs. antibiotics and orthophosphate in hospital effluents. Furthermore, indicator species analysis showed that 27% of OTUs detected in river downstream communities were indicator for urban or hospital TE sites and were absent in the river upstream. Finally, biological diatom index (BDI) calculated to evaluate the ecological status of the recipient river suggested water quality decrease linked to the release of TEs. Thus, in-depth assessment of diatom community composition using DNA metabarcoding is proposed as a promising technique to highlight the disturbing effect of pollutants in Alpine rivers.

## Introduction

Freshwater ecosystems provide important resources and services to humans. However, their sustainability is nowadays constantly affected due to their permanent application for conflicting purposes (e.g., release of wastewater and production of drinking water). WWTPs are used to reduce the release of anthropogenic pollutants into aquatic ecosystems, but pollutants cannot be completely eliminated by the treatment process (e.g., Verlicchi et al., 2015). Thus, TE usually contains a wide spectrum of highly concentrated macro- and micropollutants that may threaten ecosystems health (e.g., Verlicchi et al., 2012; Chonova et al., 2017).

Composition and toxicity level of effluents may differ strongly depending on their origin (e.g., urban, hospital, industrial), and may thereby have different impact on aquatic ecosystems (Labanowski et al., 2016). As recently shown by Chonova et al. (2016), microbial biofilm communities from urban and hospital TE sites exhibit remarkable differences in their development. Thus, the origin of pollution should not be neglected in the context of aquatic environmental monitoring. Increasing anthropogenic pressure on freshwater ecosystems imposes politically defined restrictions linked to their ecological status and functioning. In Europe, the water framework directive (WFD) aims at ensuring the maintenance of good water quality by monitoring chemical hazard substances and key aquatic indicator organisms (European Commission, 2013).

Benthic diatoms are microalgae used worldwide for water quality assessment. They are of particular interest in the context of bioassessment because of their taxonomic diversity and different species sensitivity and resistance to pollution. Diversity and community composition of diatoms adapt rapidly to the presence of chemical, physical, and biological disturbances (e.g., Stevenson and Smol, 2003). In the last decades, these features were used for the development of diatom indices that rely mainly on taxonomic composition (species or genera) obtained from microscopy counts and serve to evaluate general pollution (e.g., Coste et al., 2009; Schneider and Lindstrøm, 2009, 2011). The efficiency of DNA metabarcoding combined with high-throughput sequencing (HTS) techniques in this context was recently largely explored (e.g., Visco et al., 2015; Zimmermann et al., 2015; Vasselon et al., 2017a,b). The ability of these innovative molecular methods to provide fast, cost-efficient and reliable diatom inventories makes them promising biomonitoring tool and their application is currently being improved (e.g., Vasselon et al., 2017a, 2018).

Several studies have suggested that diatom sensitivity to environmental pressures is closely related to ecological guilds sharing functional traits (Passy, 2007; Tapolczai et al., 2016, 2017) which is also reflected by the phylogenetic tree of diatoms (Keck et al., 2016; Esteves et al., 2017). These relations give complementary insights into the health of aquatic ecosystems (Rimet and Bouchez, 2012; Larras et al., 2014) avoiding drawbacks of precise taxonomic identification (e.g., ecoregional differences in species ecological optima, taxonomic misidentification). However, the taxonomic and ecological complexity requires better understanding and further investigations.

In the context of bioassessment, diatom sensitivity is principally explored and applied in relation to nutrients and organic matter concentrations. However, the high variety of micropollutants constantly released in aquatic environments raise serious concerns about the health of these ecosystems (Schwarzenbach et al., 2006). Besides macropollutants such as nitrogen, phosphorus, and dissolved organic carbon (DOC), the possible interaction and impact of micropollutant mixtures on diatom communities should not be neglected. The sensitivity of diatoms to micropollutants (e.g., pesticides, heavy metals, and pharmaceuticals) at environmentally relevant concentrations was reported in numerous ecotoxicological studies. However, considering the large variety of diatoms and chemicals in the environment, single-species bioassays are not sufficient to provide environmentally realistic picture (Hagenbuch and Pinckney, 2012). Studies at higher complexity level are useful to fill knowledge gaps on toxic impacts. Rimet and Bouchez (2012) for example studied life-forms, ecological guilds, and cell size of diatoms in mesocosm experiments to develop a tool to assess pesticide contamination. Larras et al. (2014) found a relation between diatom sensitivity to herbicides and species’ phylogenetic position. However, assessing impacts of micropollutant mixtures on the entire diatom community in natural ecosystems, and distinguishing them from the effects induced by other factors (e.g., nutrients, flow velocity, light) remains challenging (Marcel et al., 2013).

Tapolczai et al. (2016) suggests that habitats with increased and multiple stress conditions are well adapted for studying the response of benthic diatom communities to environmental pressure. Modifications of diatom communities in rivers downstream from WWTP outputs have been already reported (e.g., Tornés et al., 2018), but community dynamics in direct response to various TEs remained thereby unknown. Despite the regular use of diatoms as biological indicators, there is a knowledge gap on their development and behavior in treated WWTP effluents. Understanding the dynamics of such communities is important to disentangle toxicity effects of pollutants mixtures and to better define a possible role of diatoms as bioindicators. These communities can be seen as a reference to better understand and evaluate the impact of TEs on recipient rivers.

In this study, we investigated community dynamics of periphytic diatoms that were exposed to hospital (H) and urban wastewater treated effluents (U), and in the recipient river up- (RU) and downstream (RD) from the WWTP output. Thereby, we aimed to (1) compare the effect of two highly polluted environments with different concentrations of pharmaceutical compounds (PhC) on diatom communities, and (2) evaluate and compare the effect of TEs’ release on benthic diatom community composition in the recipient river. Diatom communities were analyzed using a DNA metabarcoding approach. Characterization of communities regarding their diversity, taxonomic composition, phylogeny, and functional traits were used to give complimentary insights on diatoms development and behavior in habitats with different loads of pollution. Indicator species analysis (ISA) and biological diatom index (BDI) were used to evaluate the influence of TEs’ release on river communities.

## Materials and Methods

### Study Site and Biofilm Colonization Experiments

**FIGURE 1:**
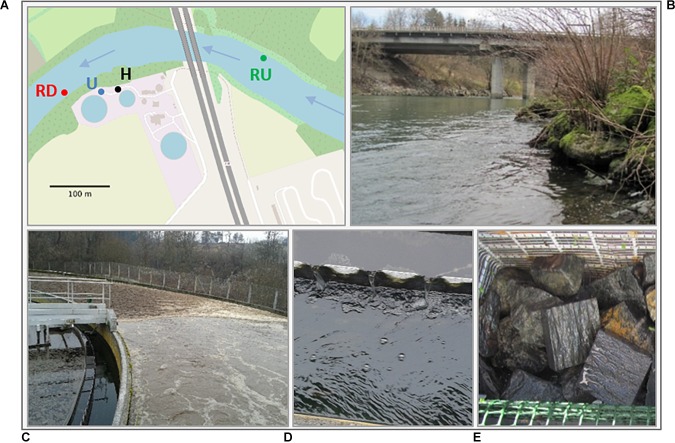
**(A)** Sampling site map (U, urban treated effluent; H, hospital treated effluent; RU, river upstream; RD, river downstream; WWTP basins are indicated with light-blue circles; approximate sampling locations are labeled with dots in the respective color); **(B)** sampling location river; **(C)** WWTP basin; **(D)** sampling location treated effluent; **(E)** blueschist stones exposed for biofilm colonization (approximate stone surface: 80 ± 20 cm^2^).

The experimental site is a pilot WWTP handling separately urban and hospital wastewater using biological treatment with conventional activated sludge system (Chonova et al., 2018b) (Figure 1A). The hospital whose effluents flow directly without special pretreatment to the WWTP has 450 beds, and the urban network includes around 20,850 inhabitants. This parallel separate treatment enables comparison of urban and hospital TEs and their effect on benthic diatoms. Treated wastewaters are discharged into the recipient River Arve. Downstream from the WWTP (ca. 18 km), water from River Arve is used for drinking water production for the city of Geneva (Switzerland), which increases the importance for the monitoring and maintenance of its high water quality.

The survey was performed between February and July 2014 in the following four locations: urban (U) and hospital (H) treated effluents and recipient river approximately 250 m upstream (RU) and 40 m downstream (RD) from the WWTP output (Figure 1). Differences in hydraulic conditions between river and TE sampling locations were observed, suggesting a higher flow velocity and turbulence in river vs. laminar conditions exhibiting at least twice lower velocity in TE sampling locations. To study the development of benthic diatom communities in each location and compare them to natural seasonal gradient, biofilm colonization experiments were done as described in Chonova et al. (2016). Metal grid-baskets with clean and previously autoclaved blueschist stones (approximate stone surface: 80 ± 20 cm^2^) were installed as substrates for natural biofilm colonization in water depth between 10 and 40 cm and were regularly inspected. Total surface of stones sampled in each location was 200 cm^2^ at least which is in accordance with the French norms used for application of WFD. After each colonization period (about 1 month), the biofilm was scraped from the stones and suspended in sterile water. Biofilm samples were collected in triplicates obtained from independent stones to ensure reliability of the experimental design. Samples were then immediately transported to the laboratory in cooling boxes for further analysis. This colonization experiment was repeated six times between February and July 2014.

### Characterization of Micro- and Macropollutants and Seasonal Conditions

Sampling sites were previously characterized in terms of nutrients (ammonium, nitrite, nitrate, orthophosphate), TSS, COD, and set of pharmaceuticals from the therapeutic classes beta-blockers (atenolol, propranolol), NSAIDs (diclofenac, ibuprofen, and ketoprofen), antibiotics (ciprofloxacin, sulfamethoxazole, and vancomycin), analgesics (paracetamol), and anticonvulsants (carbamazepine) as described in Chonova et al. (2016, 2018a). Flow-proportional 24-h sampling was performed. To obtain a representative flow-proportional sample, first, time proportional subsamples were collected every 10 min during 24 h. Secondly, subsamples from the same hour were pooled together to obtain one time-proportional sample per hour (resulting in 24 time-proportional samples of 1,020 mL in total). Finally, a fraction of each of them was combined according to the water flow rate. Nutrients, TSS and COD were measured with standard analytical methods, described by the French standard operating procedures (AFNOR, 1997). Pharmaceuticals were measured by solid-phase extraction (SPE) using hydrophilic–lipophilic balanced (HLB) columns and analyzed by HPLC–MS/MS as described by Wiest et al. (2018). For each parameter and sampling location, we calculated mean concentrations (from six monthly measurements for TE sites, and from three measurements performed in November and December 2013 and March 2014 for river sites) to give an overview of the different habitat characteristics. Measurements of hydro-meteorological conditions (river flow, urban and hospital WWTP discharge, solar irradiance, river and air temperature and precipitation) were collected as described in Chonova et al. (2018a) and means from multiple daily measurements were calculated for each colonization period to characterize seasonal trends. A theoretical dilution factor for the RD site was calculated by dividing the average river flow (m^3^.d) by the average WWTP discharge (m^3^.d^−1^).

### Metabarcoding of Diatom Samples

#### DNA Extraction and Polymerase Chain Reaction (PCR) Amplification

Total genomic DNA was isolated using Sigma-Aldrich GenElute^TM^-LPA as described in previous studies (e.g., Chonova et al., 2016). This method was recommended for diatom metabarcoding, because of its combination of various lysis mechanisms that are helpful for diatom cells opening and its ability to provide a large quantity of DNA (Vasselon et al., 2017a).

A 312 bp fragment of the *rbc*L plastid gene, recommended as DNA barcode for diatom metabarcoding (Kermarrec et al., 2013), was amplified using Takara LA Taq^®^ polymerase and the primer pairDiat_rbcL_708F (AGGTGAAGTTAAAGGTTCATACTTDAA) (Stoof-Leichsenring et al., 2012) and R3 (CCTTCTAATTTACCAACAACTG) (Bruder and Medlin, 2007). Each PCR amplification mix (total volume of 25 μL) contained 1 μL of 25 ng DNA template, 0.75 U of Takara LA Taq^®^ polymerase, 2.5 μL of 10× PCR Buffer, 1.25 μL of 10 μM of each primer, 1.25 μL of 10 g/L BSA, 2 μL of 2.5 mM dNTP, and ultrapure water to complete. For each set of reactions, a negative control was included. PCR reaction conditions were initiated by a denaturation step at 95°C for 15 min followed by a total of 30 cycles of 95°C for 45 s (denaturation), 55°C for 45 s (annealing), and 72°C for 45 s (final extension) (Vasselon et al., 2017a).

#### Genomic Libraries Preparation

Genomic libraries for HTS were prepared from PCR amplicons of the *rbc*L 312 bp fragment from four independent PCR reactions per sample as described in Vasselon et al. (2017a). Briefly, PCR amplicons were cleaned with Agencourt AMPure beads (Beckman Coulter, Brea, CA, United States). Quality and quantity of purified products were measured with 2200 Tape Station (Agilent Technologies, Santa Clara, CA, United States). Tags were added to each amplicon and libraries were prepared using the NEBNext^®^ Fast DNA Library Prep set for Ion Torrent^TM^ (BioLabs, Ipswich, MA, United States) and A-X tag adapter provided by Ion Express^TM^ Barcode adapters (Life Technologies, Carlsbad, CA, United States). Finally, all libraries were pooled at a final concentration of 100 pM and sequenced using an Ion 316^TM^ Chip Kit V2 (Life Technologies, Carlsbad, CA, United States) on a PGM Ion Torrent sequencer by the “Plateforme Genome Transcriptome” (PGTB, Bordeaux, France).

#### Sequence Data Processing

The sequencing platform performed demultiplexing and adapter removal. Data were provided in form of separate fastq files for each sample. Bioinformatic processing was performed with Mothur Software (Schloss et al., 2009) as proposed by Vasselon et al. (2017a). Low quality sequences (read length below 250 bp, Phred quality score below 23 over a moving window of 25 bp, more than one mismatch in the primer sequence, homopolymer over 8 bp or presence of ambiguous base) were removed and obtained quality filtered sequences from all samples were analyzed jointly. Removal of chimera using Uchime algorithm (Edgar et al., 2011) was performed after pre-clustering (applied for denoising of sequencing error based on cluster generation by reads with only one nucleotide difference). Subsequently, taxonomic affiliation of DNA reads was performed using Rsyst::diatom barcoding library v4 (Rimet et al., 2016) and the naïve Bayesian method (Wang et al., 2007) with a confidence score threshold of 85%. All reads assigned to groups different from Bacillariophyta (diatoms) were excluded from further analyses. Similarity distance matrix based on pairwise distances between aligned reads (algorithm proposed by Needleman and Wunsch, 1970) was computed and used to cluster reads in operational taxonomic units (OTUs) with the furthest neighbor algorithm (95% similarity level as proposed by Mangot et al., 2013). Singleton removal and sample size normalization (to the smallest read abundance obtained among all samples) were performed and OTUs were taxonomically assigned based on consensus taxonomy of reads using confidence threshold of 80%. Taxonomy of abundant OTUs (above 100 reads), that were not precisely affiliated with the naïve Bayesian method, were verified with Rsyst::diatom blast to confirm their belonging to Bacillariophyta.

### Data Analysis

Quantitative (normalized abundances) and qualitative (presence–absence) matrices of OTUs were used to analyze diatom communities. All statistical analyses were performed using the free and open source R software (3.4.4, R development core team).

#### Diatom Richness, Diversity, and Community Structure

Chao1 richness and Simpson diversity were calculated for all sampling dates and locations. NMDS was performed with Mothur Software on Bray–Curtis dissimilarity matrix to compare diatom communities. Differences between sites and seasons were tested with PERMANOVA (*p* < 0.05) (Anderson, 2001; McArdle and Anderson, 2001) made using the vegan package (Oksanen et al., 2017).

#### Community Changes Linked to Environmental Factors

Redundancy analysis (RDA) was performed to infer the relationship between environmental factors and diatom communities from urban (U) and hospital (H) TE sites (vegan package, Oksanen et al., 2017). Prior to the analysis, the biological data (normalized quantitative OTU matrices) were Hellinger-transformed as recommended for linear ordination methods (Legendre and Gallagher, 2001). To reduce the impact of high correlations between environmental variables, an approach based on principal component analysis (PCA) was applied. Three pairs of highly correlated variables (*r* > 0.7) were identified: air temperature and irradiance (Temp.Irr), beta-blockers and NSAIDs (BB.NS), antibiotics and orthophosphate (ATB.PO4) and three separate PCAs were performed for each of the pairs. The first axis of each PCA (accounting for 98%, 92%, and 89% of the total variability for Temp.Irr, BB.NS, and ATB.PO4, respectively) was used in subsequent analysis as a synthetic variable representative for the variability of both variables.

In a next step, forward selection procedure was ran on the RDA performed on OTUs and the environmental variables ammonium, nitrite, nitrate, anticonvulsants, analgesics, TSS, COD, BB.NS, ATB.PO4, and Temp.Irr to obtain the most parsimonious RDA model with a reduced number of variables. The effect of time dependence was thereby considered by including the sampling month as a covariate. RDA was performed with the selected parameters, VIFs were verified and the significance of each variable and of the whole model was tested with permutation tests (999 permutations, *p* < 0.05).

Finally, variation partitioning based on multiple partial RDAs (pRDAs) was performed to quantify and test the proportion of biological variability explained by each of the selected variables (Borcard et al., 1992). The variation explained by each fraction was reported using unbiased adjusted *R*^2^ (Peres-Neto et al., 2006). Permutation test (999 permutations, *p* < 0.05) was used to test the significance of each fraction.

#### Relative Genera Abundances and Ecological Guild Classes

Relative abundances of OTUs affiliated on genus level and of ecological guild classes were calculated from means of sample triplicates and presented in barplots for each sampling location and period to study spatial and temporal diatom community dynamics. Ecological guilds were based on the original classification by Passy (2007) including the modifications proposed by Rimet and Bouchez (2012).

#### Phylogeny and Indicator Species Analysis of OTUs

A phylogeny of sequences from the final list of OTUs was reconstructed. First, the OTU sequences were aligned against the *rbc*L sequences of the R-Syst::diatom database using the muscle algorithm (Edgar, 2004). Second, OTUs were inserted in a reference phylogeny of 604 species with the evolutionary placement algorithm (Berger et al., 2011) implemented in RAxML (Stamatakis, 2014). Finally, the tree was dated in relative time using PATHd8 (Britton et al., 2007).

Indicator species analysis was performed to identify OTUs that discriminate diatom communities in U, H, and RU locations. Indicator values based on relative abundance of OTUs were calculated following the method proposed by Dufrene and Legendre (1997) implemented in the labdsv package (Roberts, 2007). The indicator values of each OTU were calculated based on abundance values and relative frequency of occurrence and tested using a randomization procedure (999 permutations, *p* < 0.05). Each significant OTU was considered to be indicator of the location for which it exhibited the highest indicator value. Thus, a list of indicator OTUs was obtained for each location. Subsequently, indicator OTUs were denoted in the phylogenetic tree with location-specific colors for U (blue), H (black), and RU (green). Remaining non-significant OTUs were denoted with gray. Significant indicator OTUs of all three locations that appeared in RD were also labeled with the respective location-specific colors. Proportion of U, H, and RU indicator OTUs in RD communities was calculated (considering number of OTUs and DNA read abundances) to evaluate the influence of each location-characteristics on RD communities.

#### Biological Diatom Index

Biological diatom index is a biotic index calculated after morphological identification of diatom species present in natural biofilms. It is routinely applied for water quality assessment of running waters in France and is used in the context of the WFD (Coste et al., 2009). In this study, OTUs were affiliated on species (or genus when species not available) level and their normalized quantitative matrices, based on read numbers, were used to calculate molecular BDI for each sampling location and period and to evaluate thereby the effect of TEs on water quality in the recipient river. BDI calculation was done with OMNIDIA 5 software (Lecointe et al., 1993).

## Results

### Habitat Description

Principle major differences regarding nutrients and pharmaceuticals were found between sampling locations, especially distinguishing TE sites from river sites. Nutrients and PhC were clearly higher concentrated in the TEs (Table 1). Comparing urban and hospital effluents, the clearest trends were observed for orthophosphate and antibiotics with significantly higher concentrations in H, and beta-blockers, NSAIDs and ammonium – in U (paired Wilcoxon test, *p* < 0.05). In river locations, concentrations of PhC (especially antibiotics, NSAIDs and anticonvulsants) and orthophosphate showed rather increasing trend in RD compared to RU, which was not noticeable for ammonium, nitrite, nitrate, and TSS (Chonova et al., 2018a). During the observation period solar irradiance, air temperature and river flow increased from winter to summer. Threefold increase of the theoretical dilution factor for WWTP discharge was observed from winter to summer (Table 2).

**Table 1:** Mean concentrations of nutrients and pharmaceuticals in urban (U) and hospital (H) treated effluents and Arve river up- (RU) and downstream (RD) from the WWTP Output (standard deviation in brackets).

	U	H	RU	RD
Ammonium (mg⋅l^−1^)	4 (3.7)	0.3 (0.4)	0.24 (0.06)	0.06 (0.02)
Nitrite + nitrate (mg⋅l^−1^)	16.5 (9.6)	65.6 (58.7)	1.2 (0.3)	0.8 (0.09)
Orthophosphate (mg⋅l^−1^)	2.3 (1)	8.9 (1.5)	0.02 (0.01)	0.04 (0.01)
COD (mg⋅l^−1^)	22.8 (5.1)	23.9 (2.9)	-	-
TSS (mg⋅l^−1^)	4.5 (2.5)	5.3 (1.7)	3.5 (0.7)	3.3 (2.5)
NSAIDs (μg⋅l^−1^)	1 (0.4)	0.14 (0.04)	0.0004 (0)	0.0301 (0.013)
Beta-blockers (μg⋅l^−1^)	0.5 (0.19)	0.2 (0.06)	0.0036 (0.0047)	0.0119 (0.0071)
Antibiotics (μg⋅l^−1^)	0.09 (0.06)	2.6 (1.5)	0.0025 (0.0001)	0.0054 (0.0022)
Anticonvulsant (μg⋅l^−1^)	0.5 (0.12)	0.6 (0.19)	0.002 (0.0027)	0.0118 (0.0055)
Analgesic (μg⋅l^−1^)	0.4 (0.31)	0.9 (1.3)	0.138 (0.0141)	0.1672 (0.0648)
Total PhC (μg⋅l^−1^)	2.5 (0.6)	4.5 (2.6)	0.1465 (0.0122)	0.243 (0.120)

*PhC, pharmaceutical compounds; COD, chemical oxygen demand; TSS, total suspended solids; NSAIDs, non-steroidal anti-inflammatory drugs.*

**Table 2:** Mean values calculated from multiple daily measurements of solar irradiance, river temperature, precipitation, river flow, and WWTP discharge for each colonization period (standard deviation in brackets; data sources: INRA meteorological station^∗^, federal office of environment of Switzerland^∗∗^ and SIPIBEL observatory).

	February	March	April	May	June	July
Air temperature (°C) ^∗^	4.3 (1.4)	6.8 (2.2)	10.7 (2.4)	12 (2.1)	17.2 (3.4)	18 (2.2)
Solar irradiance (MJ⋅m^−2^) ^∗^	5.2 (3.1)	11.4 (4.3)	16.9 (5.7)	17.4 (5.6)	23.9 (6.8)	20.6 (8)
Precipitation (mm⋅day^−1^) ^∗^	4.9 (7.1)	2 (6)	0.6 (1.9)	3.3 (4.9)	2.7 (8.4)	5.1 (7.7)
River temperature (°C) ^∗∗^	5.6 (0.8)	7.2 (0.8)	9.1 (0.9)	9.9 (1)	11.5 (0.8)	11.9 (0.9)
River flow (m^3^⋅s^−1^) ^∗∗^	61 (23)	59.6 (15.9)	67 (18.9)	83.4 (16.9)	92.1 (24.4)	101.4 (53.1)
Hospital discharge (m3⋅day^−1^)	152 (31)	151 (27)	126 (26)	129 (26)	136 (25)	147 (33)
Urban discharge (m3⋅day^−1^)	7965 (2372)	6163 (1686)	4223 (501)	4542 (573)	4668 (1478)	4236 (1283)
Dilution factor for WWTP discharge	642 (107)	872 (317)	1346 (374)	1545 (283)	1706 (372)	1909 (480)

### High-Throughput Sequencing

We obtained 3,760,984 reads from the HTS, with an average of 59,698 reads per sample and average length of 312 base pairs. After the bioinformatics filtration steps, 1,357,398 reads were retained and were clustered into 1,121 OTUs (Supplementary Table S1). All samples were subsampled to 11,306 reads (lowest read abundance obtained for a sample) to allow inter-sample comparison which resulted in total of 1,076 OTUs with minimum of 103 and maximum of 301 OTUs per sample. R-Syst::diatom v4 database enabled taxonomic affiliation of 103 different species from 48 different genera. 81% of the OTUs (90% of the reads) were affiliated at family level, 75% of the OTUs (85% of the reads) – at genus level and 57% of the OTUs (70% of the reads) – at species level. Those results are in consistence with other studies (e.g., Vasselon et al., 2017b). Most of the unclassified reads were found in H – a location that represented the most extreme environment in terms of concentrations of micro- and macropollutants. However, results from R-Syst::diatom blast confirmed that unclassified OTUs belong to the group Bacillariophyta and were not misclassified.

### Diatom Community Changes and the Role of Micro- and Macropollutants

**FIGURE 2:**
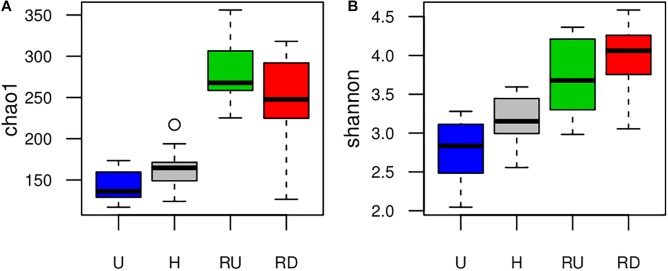
Boxplots presenting variation in **(A)** Chao1 richness and **(B)** Shannon diversity calculated from HTS-OTUs of benthic diatoms developed in urban (U) and hospital (H) treated effluent sites and river sites up- (RU) and downstream (RD) from the WWTP output.

Boxplots on Figure 2 compare richness (Chao1) and diversity (Shannon) of benthic diatom communities between sampling locations and suggested increased richness and diversity in the river compared to the TEs. Higher richness and diversity were observed in H than in U. Comparing river locations, alpha diversity changed depending on the method – Chao1 reported higher richness in RU, and Shannon suggested tendency for higher diversity in RD. Seasonal trends of diatom richness and diversity were not observed.

**FIGURE 3:**
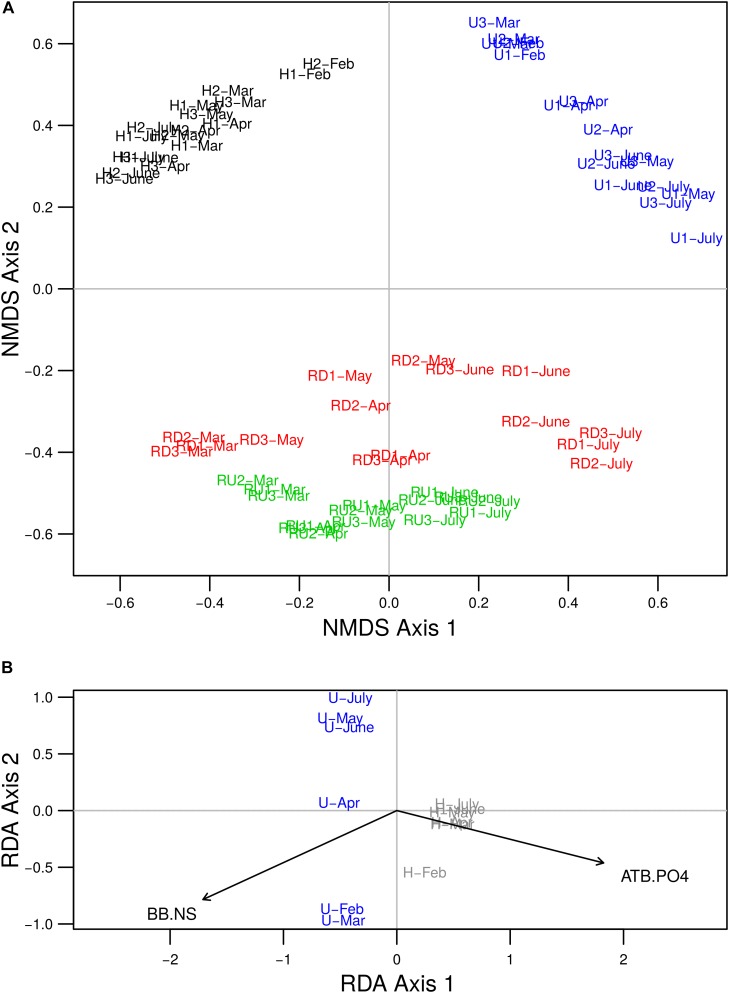
**(A)** NMDS two-dimensional plot of observed similarities between *rbc*L OTU profiles of benthic diatom communities of urban (U) and hospital (H) treated effluents and river sites up- (RU) and downstream (RD) from the WWTP output (stress value = 0.22). **(B)** Biplot from redundancy analysis based on diatom OTU profiles for periphytic samples from TE sites (U and H), and the variables ATB.PO4 and BB.NS chosen with forward selection procedure. Colors delineate sampling locations: U (in blue), H (in black or gray), RU (in green) and RD (in red). Numbers (1, 2, and 3) in the sample name denote triplicates.

Non-metric multidimensional scaling analysis of quantitative OTU matrix showed that diatom communities were mainly grouped by sampling location (Figure 3A). Significant differences between U and H communities were represented on the first axis, and between TE sites and river sites on the second axis (PERMANOVA, *p* < 0.05). RD communities were more similar to U and H than RU communities. As secondary shaping factor, seasonal changes were visible for all sampling locations.

Redundancy analysis was performed to explore the relationship between diatom community structure and environmental variables measured in TEs. Forward selection procedure identified that the best parsimonious model includes the variables ATB.PO4 (representing 89% of the common variability of antibiotics and orthophosphate) and BB.NS (representing 92% of the common variability of beta-blockers and NSAIDs) as the most important for diatom community changes (Figure 3B). RDA model including ATB.PO4 and BB.NS was significant and the two variables tested separately were significant to the model (*p* < 0.05). The low VIF values (VIF < 3) suggested that there is no issue with collinearity. The first two axes of the model accounted for 61% and 3% of the variability in diatom community structure, respectively, when considering the effect of time dependence as covariate. The first axis defined a local gradient between the two basins, where diatom community development in U was rather linked to higher concentrations of beta-blockers and NSAIDs and in H – to antibiotics and orthophosphate. The second axis explained a low proportion of the variance only. Variation partitioning revealed that the effect explained by ATB.PO4 and BB.NS separately remained significant (*p* < 0.05) and it accounted for 17% and 11% of the total variability for ATB.PO4 and BB.NS, respectively. The variance shared between the two variables was 36%.

### Spatial and Temporal Variations in Diatom Genera and Ecological Guild Classes

**FIGURE 4:**
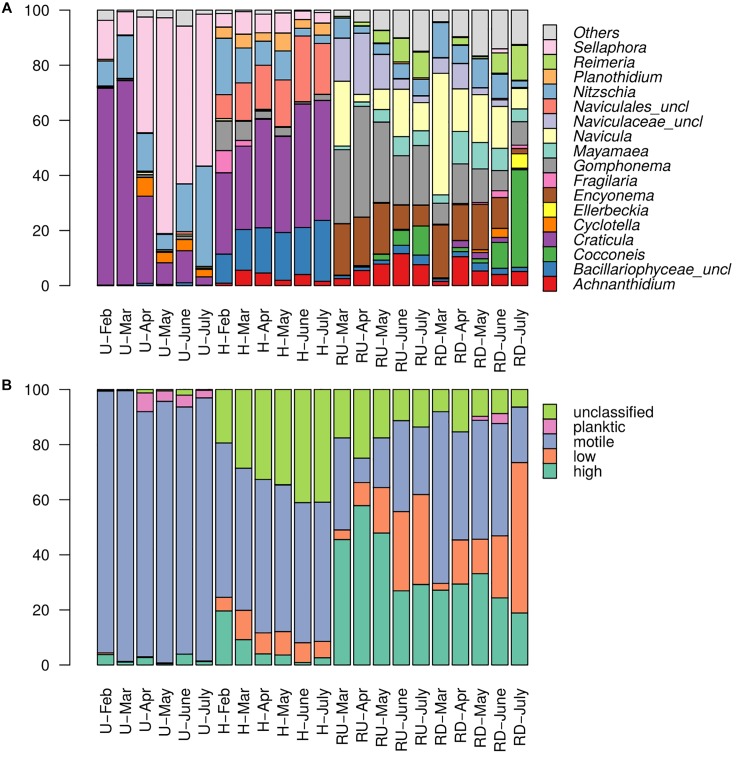
Relative abundances of diatom **(A)** genera and **(B)** ecological guild classes (in %) in each sampling site and period. “Others” – sum of all genera representing less than 5%.

Relative abundances of diatom genera (a) and ecological guild classes (b) per sampling site and period are presented in Figure 4. Planktic species were rare in the biofilms. Clear dichotomy in ecological guild classes was found between TE sites and river sites.

At TE sites mainly the development of polysaprobic species (e.g., *Sellaphora*, *Craticula*) was observed. Motile groups (represented mainly by the genera *Craticula*, *Sellaphora*, and *Nitzschia*) dominated in treated wastewater with relative abundance up to 95% in U. Their dominance slightly decreased in H, where motile guild was partially replaced by low- (*Achnanthidium* and *Planothidium*) and high-profile groups (*Gomphonema*). Seasonal trends were observed in U for the genus *Craticula* and *Sellaphora*, as *Craticula* was dominating in colder months (February and March) with ca. 70% relative abundance but was replaced by *Sellaphora* in warmer months. In H, *Nitzschia, Gomphonema*, and *Fragilaria* exhibited slightly higher relative abundance in colder months and decreased in summer.

In river communities, genera relative abundances were more evenly distributed and low- and high-profile species were better represented (especially in RU). Importance of *Craticula* and *Sellaphora* decreased and motile groups were rather represented by *Mayamaea*, *Navicula*, and *Nitzschia*. Overall, in the river, groups with oligotrophic and oligosaprobic (e.g., *Achnanthidium*, *Encyonema*) preferences were favored. This trend was clearly stronger expressed in the river upstream from the WWTP output. Seasonal trends were observed for low- and high-profile diatoms. Low-profile groups (mainly *Achnanthidium*, *Cocconeis*, and *Reimeria*) were more abundant in warmer months, while increase in high-profile diatoms (mainly *Encyonema* and *Gomphonema*) was observed rather in colder months, especially upstream from the WWTP.

### Location-Specific Indicator OTUs

**FIGURE 5:**
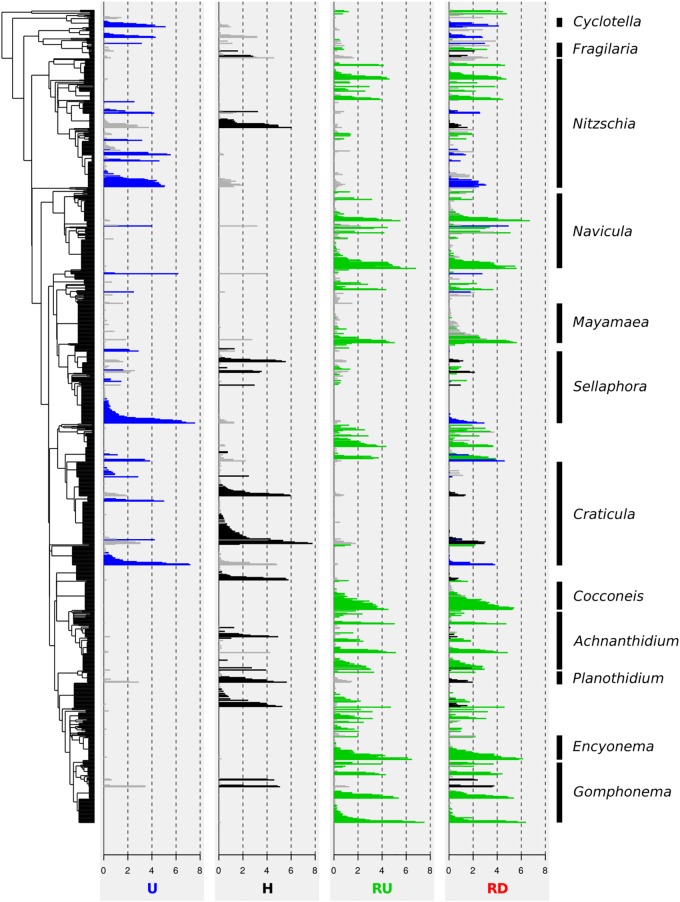
Phylogenetic tree of 1,076 OTUs (log-transformed relative average abundance) of benthic diatoms from urban (U) and hospital (H) treated effluent sites and river sites upstream (RU) and downstream (RD) from the WWTP output. Colors delineate indicator OTUs for each location: U (in blue), H (in black), and RU (in green).

Abundances of OTUs were calculated as mean of all samples for each location and are presented (log-transformed) in Figure 5. Indicator OTUs of U, H, and RU (represented in blue, black, and green on the figure, respectively) were located in different parts of the phylogenetic tree. Indicator OTUs of U belonged mainly to *Nitzschia*, *Sellaphora*, and *Craticula*, whereas a large phylogenetic clade comprising *Cocconeis*, *Achnanthidium*, *Planothidium*, *Encyonema*, and *Gomphonema* was hardly present in this location. By contrast, many indicator OTUs belonging mainly to *Achnanthidium*, *Planothidium*, and *Gomphonema* appeared again in H, and became even more important for RU. Indicator OTUs of RU were detected throughout the phylogenetic tree, except for *Craticula* and *Sellaphora*. These results confirmed general trends revealed with the relative abundance of genera.

Figure 5 clearly illustrates that most of the OTUs found in RD corresponded to indicator OTUs of RU (53% of the OTUs, corresponding to 88% of the DNA reads). However, numerous indicator OTUs of U or H that were not present in RU appeared in RD (e.g., *Craticula*, *Sellaphora*, and *Nitzschia*). This proportion was 14% of the OTUs (corresponding to 7% of the reads) for U, and 13% of the OTUs (corresponding to 2% of the reads) for H.

### Biological Diatom Water Quality Index

**FIGURE 6:**
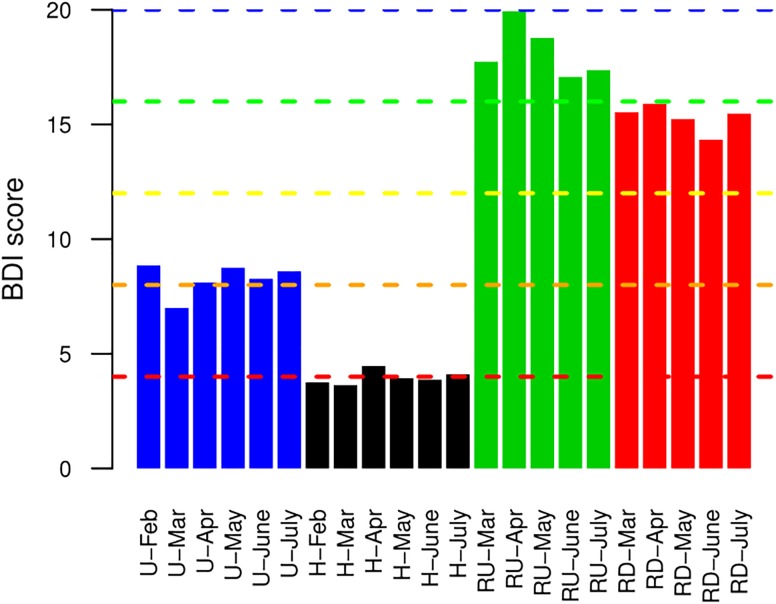
Biological diatom water quality index (BDI) calculated for benthic diatoms from urban (U) and hospital (H) treated effluent sites and river sites up- (RU) and downstream (RD) from the WWTP.

Biological diatom index was calculated from molecular data and represented in Figure 6. As expected, BDI suggested that water quality was lower at TE sites compared to the recipient river. Lowest values were observed for H (quality between “bad” and “poor”), followed by U (between “poor” and “moderate”). At river sites, BDI revealed “high” quality status in RU and decrease from “high” to “good” in RD.

## Discussion

The development and behavior of diatom communities in response to TEs remain poorly documented. Microalgae have been previously associated to wastewater treatment due to their ability to assimilate nitrogen and phosphorus and to bind heavy metals (e.g., Congestri et al., 2005). In this context, in recent studies Ghosh and Love (2011) investigated algal assemblages from the water column of a secondary WWTP tank, and Congestri et al. (2005) reported communities developed on tank walls and artificial substrata in WWTP sedimentation tanks. In such highly polluted environments, diatoms represent one of the most abundant groups among algae (e.g., Ghosh and Love, 2011). Although diatoms are regularly used for biomonitoring and their sensitivity to micropollutants is well known, species composition and structural dynamics in WWTPs and their TEs have rarely been documented in this context. The present study reports for the first time the reliability of DNA metabarcoding to detect spatial and temporal responses of diatom assemblages to high environmental pressure in urban and hospital TEs and contributes to our understanding of the effluents’ impact on community dynamics in the recipient aquatic environment.

Parallel analysis of nutrients, PhC and seasonal parameters provided essential information about the characterization of different habitats and confirmed higher presence of pollutants in the TEs compared to the river. As expected, comparison of U and H showed that hospital TEs contained higher total concentrations of PhC (especially antibiotics) and phosphate caused by the regular use of PhC and specific detergents in the hospital. However, better removal efficiency of NSAIDs and beta-blockers in the hospital treatment process might have resulted in their higher concentrations in urban TEs (Chonova et al., 2016). Release of WWTP effluents in the recipient river led to increased concentrations of PhC (especially NSAIDs, antibiotics, and anticonvulsants) and phosphate downstream from the TEs output. In contrast nutrients such as ammonium, nitrate, nitrite, and TSS did not show such up – to downstream gradient. Temperature, solar irradiance and river flow (caused by the melting of glacier) followed the usual seasonal gradient with increase in summer. No extreme water level changes or flood events were observed during the study experimental period.

### Composition of Diatom Communities Linked to Habitats Characteristics

Diversity and community structure of benthic diatoms exhibited clearly stronger local changes than seasonal ones, which highlights the relevance of location-specific factors (e.g., nutrients and PhC) over seasonal factors (e.g., temperature and solar irradiance) (Figure 2, 3). The high relevance of nutrients in structuring diatom communities has been well studied (e.g., Patrick, 1961; Lange-Bertalot, 1979; Larson and Passy, 2012; Marcel et al., 2013). Several studies also show significant effect of PhC (e.g., NSAIDs, beta-blockers, antibiotics) and other micropollutants (pesticides, heavy metals, etc.) on benthic diversity and community structure (e.g., Hagenbuch and Pinckney, 2012; Larras et al., 2013; Corcoll et al., 2014). Temperature, solar irradiance and flow velocity (for river sites) were previously shown to be crucial factors for the development of benthic diatom communities (Lange et al., 2011; Villeneuve et al., 2011; Larras et al., 2013). However, in this study they remained of secondary importance. Such primary local and secondary seasonal community dynamics at the same study site were reported for bacterial biofilms after short- and long-term biofilm colonization periods, where both nutrients and PhC played a key role in the shaping of the community structure (Chonova et al., 2016, 2018a).

#### Response of Diatoms to Pollutants in Urban and Hospital Treated Effluents

Communities developed at TE sites and at river sites differed strongly regarding their richness and diversity which reflected the contrasting ecological differences between the two systems (Table 1). Considerably lower diatom richness and diversity were observed at TE sites which were the most severely disturbed and showed higher concentrations of micro- and macropollutants compared to the natural river system (Figure 2). Such low diatom diversity was also reported in studies investigating algal communities in WWTP clarification tanks and is probably linked to the high anthropogenic pressure which leads to the elimination of less resistant species (e.g., Sládečková et al., 1983; Congestri et al., 2005). However, restricted colonization possibilities in these “artificial systems” offering limited microhabitats compared to natural environments may also affect the number of species. This question needs to be further investigated applying translocation experiments as described for example by Proia et al. (2013b).

Diatom community structure at TE sites also differed from river sites regarding HTS-OTUs, genera taxonomic level and ecological guild classes (Figure 3, 4). These trends allowed to better understand the community dynamics linked to the ecological preferences of diatoms, and clearly showed that community adaptations are in accordance with habitat characteristics. In general, TE sites characterized by higher anthropogenic pressure and lower water velocity and turbulence, were dominated by few genera with polysaprobic preferences and well-developed raphe system belonging to the motile guild (e.g., *Craticula*, *Nitzschia*, *Sellaphora*, etc.). Some of these genera (*Craticula*, *Nitzschia*, *Sellaphora*, and *Gomphonema*) were also previously reported as dominant in WWTP tanks (Congestri et al., 2005). As described by Passy (2007) and Rimet and Bouchez (2012), the motile guild included free-moving species with tolerance to high nutrient concentrations and low resistance to flow velocity. Higher tolerance of motile species to micropollutants (e.g., herbicides and fungicides) has also been reported previously (Rimet and Bouchez, 2011). This resistance to pollution is linked to the ability of motile diatoms to optimize their position in the biofilm and thereby avoid disturbances (Lengyel et al., 2015). The adaptations of motile groups to survive and reproduce in highly contaminated environments explains their dominance over other guilds in TEs. Dominance of raphid taxa in the TEs was also reported previously in WWTPs (Congestri et al., 2005; Ghosh and Love, 2011) and may be explained by their higher resistance to micropollutants which may lead to the replacement of higher sensitive centric and araphid taxa (e.g., Larras et al., 2014 for pesticides).

According to Ghosh and Love (2011), algal diversity and composition may differ strongly between different WWTPs. Here, we also found differences when comparing separately treated urban and hospital effluents, despite their close geographic proximity. Hence, these differences are probably rather linked to the origin-specific effluent composition than to the spatial effect on colonization possibilities. Interestingly, lower richness and diversity was observed in U, where concentrations of total nutrients and PhC were lower (Figure 2 and Table 1). Differences between U and H communities were also observed on OTU and genera level, and to lower extent – regarding functional traits (Figure 3, 4). Urban communities in colder months (February and March) were dominated by *Craticula*, which was replaced by *Sellaphora* in warmer months. Both species are symmetrical biraphid diatoms and exhibit similar trophic preferences since they tolerate nutrient rich environments. Their similar characteristics may lead to competition resulting in suppression of *Craticula* in warmer – and *Sellaphora* in colder months. *Nitzschia* was also regularly found in U, but did not exhibit such strong seasonal variations. In hospital communities, low- (*Achnanthidium* and *Planothidium*) and high-profile groups (*Gomphonema*) showed higher relative abundances compared to U (Figure 4B). However, motile groups (*Craticula*, *Nitzschia*, and *Sellaphora*) remained dominant. Regarding seasonal trends, *Nitzschia, Gomphonema*, and *Fragilaria* exhibited slightly higher relative abundance in colder months, but genera composition in H remained generally stable between seasons.

Aiming to better comprehend community changes and to categorize the importance of nutrients, pharmaceuticals, and seasonal conditions, diatom community dynamics in U and H were further studied on OTU level with RDA. Forward selection procedure defined beta-blockers, NSAIDs, antibiotics and phosphate as the most important factors explaining a significant part of the diatom OTU variability (64%). Higher concentrations of beta-blockers and NSAIDs (accounting for 11% of the biological variability) were rather linked to urban communities, while antibiotics and phosphate (accounting for 17%) – to hospital ones. Beta-blockers and NSAIDs, in the same concentration range as reported in U, may inhibit algal photosynthesis processes (Bonnineau et al., 2010) and change benthic community structure (Corcoll et al., 2014). Lower diversity in U may therefore be linked to the influence of these PhC. Other studies showed that NSAIDs may change phosphatase activity (Proia et al., 2013b) and reduce photosynthesis (Ding et al., 2017). Negative effect of antibiotics on benthic communities has also been reported (e.g., Proia et al., 2013b) and associated with reduction or elimination of diatom mobility (Hagenbuch and Pinckney, 2012) and inhibition of diatoms growth due to reduced photosynthesis (Pinckney et al., 2013; Guo et al., 2016). Hence, mobility reduction in H, where antibiotic concentrations were more than 20 times higher than in U, may have led to discrimination of motile diatoms, favoring development of low- and high-profile species. Furthermore, effect of antibiotics on bacterial communities may lead to indirect changes in diatom assemblages (e.g., Windler et al., 2015). The remaining PhC (paracetamol and carbamazepine) were excluded by the forward selection procedure in the course of RDA. However, considering the high concentrations of the analgesic paracetamol and the anticonvulsant carbamazepine, we cannot exclude their possible effect on diatom communities. Indeed, paracetamol may also impact photosynthesis and cause algal growth inhibition (Proia et al., 2013b). The presence of non-measured PhC, metabolites, pesticides, heavy metals and detergents may also have played a role in the shaping of diatom communities (e.g., Morin et al., 2009; Ricciardi et al., 2009). The mixture of highly concentrated pollutants found in threated effluents may exhibit additive, synergistic and antagonistic effects on benthic communities (Hagenbuch and Pinckney, 2012; Verlicchi, 2018). Such complex cocktail effects may strongly vary depending on the present compounds and the microbial communities. Hence, single-compound/species studies are not sufficient to make meaningful predictions of the ecological impact of PhC and potential toxic effects of “micropollutant cocktails” need to be better understood (Hagenbuch and Pinckney, 2012).

#### Dynamics of Benthic Diatoms in Recipient River

River habitats (RU and RD) in this study are characterized as natural aquatic ecosystem and exhibit lower anthropogenic pressure and higher flow velocity and turbidity (particularly in spring and summer when river flow increases due to the snow and glacier melting) compared to TE sites. In the river, species with oligosaprobic preferences were generally favored and the development of diverse diatom communities and higher evenness between genera was facilitated. Consequently, lower dominance of motile groups (here represented mainly by *Mayamaea*, *Navicula*, and *Nitzschia*) and better growth of low- and high-profile groups was observed. In both river locations, higher prevalence of low-profile genera (represented mainly by *Achnanthidium*, *Cocconeis*, and *Reimeria*) was observed in summer (June and July). This can be explained by their better adaptation to resist to high current velocity and strong water turbulence that increase in these months. In contrast, high-profile groups (represented mainly by *Encyonema* and *Gomphonema*), that are not well adapted to high velocity, developed better in winter (Passy, 2007).

Benthic diatom assemblages in the river up- and downstream from the WWTP effluent were compared to evaluate the effect of TEs on the recipient aquatic environment. Regarding richness and diversity, we observed inconsistency between Chao1 (lower richness in RD) and Shannon (lower diversity in RU) (Figure 2). This difference may be linked to the increase in anthropogenic pressure in RD resulting in loss of rare species (ca. 20% less OTUs with <3 reads) (Proia et al., 2013a). Biggs (2000) observed that under certain circumstances, major species replacements with increasing eutrophication may also result in increase of species richness. Contrasting conclusions have been reported for the response of diatom richness and diversity to presence of micropollutants as rather a decrease in the presence of micropollutants (mainly herbicides and heavy metals) was observed (e.g., Genter and Lehman, 2000; Sabater, 2000; Morin et al., 2009; Ricciardi et al., 2009), while others were unable to detect such relation (e.g., Hirst et al., 2002; Marcel et al., 2013). Richness and diversity indices are not always relevant to assess waterbodies pollution levels (Ricciardi et al., 2009; Pandey et al., 2017). They reduce community information to a single number, which leads to huge loss of essential information about various crucial community characteristics (e.g., taxonomic structure, functional traits, phylogenetic relations, etc.) (Biggs, 2000).

Changes in community structure between river sites reflected the pollution induced by the TEs discharge that resulted in increasing trends of phosphate, NSAIDs, antibiotics and anticonvulsants downstream from the WWTP output. Species with oligotrophic and oligosaprobic preferences and high-profile taxa (that are more sensitive to contamination of micropollutants, Marcel et al., 2013) were rather disadvantaged downstream from the WWTP comparing to upstream. In RD, these were replaced by motile groups, reflecting the increase in anthropogenic pollution (Rimet and Bouchez, 2011). Motile groups are usually less resistant to current velocity (Passy, 2007) and it was previously reported that they develop better in winter (Stenger-Kovács et al., 2013). However, in this study they exhibited such seasonal trend in RD only, which may be rather linked to the lower dilution factor of TEs in winter, potentially leading to stronger impact on river diatoms during winter.

Chronic input of trace emerging contaminants may in general lead to decrease in biofilm biomass and primary productivity and may thereby influence the ecology of benthic diatom assemblages (Hagenbuch and Pinckney, 2012). Such changes in more sensitive systems may impair higher trophic levels and lead to important alterations in river ecosystem functioning (Pinckney et al., 2013). Long-term exposure to contaminants may finally result in more tolerant assemblages (e.g., Corcoll et al., 2014). Results presented here are helpful to follow the changes on natural diatom communities potentially caused by the release of WWTP effluents. However, further studies are needed to better comprehend the cocktail effect of PhC and other micropollutants on benthic diatoms in aquatic environments. When studying such relationships, the potential effect of confounding factors (e.g., eutrophication levels, seasonal conditions) has to be carefully considered (Marcel et al., 2013).

### Tracking the Effect of Urban and Hospital Treated Effluents on River Communities

#### Indicator Species Analysis

Indicator species analysis in combination with phylogenetic tree representation was helpful to better understand the effect of TEs on diatom assemblages and disentangle community changes in natural aquatic environment receiving urban and hospital TEs. Clades of phylogenetically close indicator OTUs behaved similarly and appeared simultaneously in the different habitats. Such patterns were expected from highly similar OTUs, as the clustering method applied here may generate several OTUs belonging to the same species. However, this trend also appeared on a larger scale in the phylogeny. Indicator OTUs from a large clade including mainly *Cocconeis*, *Achnanthidium*, *Planothidium*, *Encyonema*, and *Gomphonema* were hardly present in U, and those from a clade including mainly *Navicula* and *Mayamaea* were not found in H. Such phylogenetically related groups are likely to share similar characteristics like guild classes and ecological preferences (Keck et al., 2016). *Cocconeis*, *Achnanthidium*, *Planothidium*, *Encyonema*, and *Gomphonema* all possess a developed raphid system and belonged to the high- or low-profile guilds. In contrast, *Navicula* and *Mayamaea* are part of the motile guild. Indicator OTUs discriminating RU were detected throughout the phylogenetic tree. Entire clades from genera highly dominant at TE sites (e.g., *Craticula*, *Sellaphora*, and partially *Nitzschia*) were hardly found in RU implying their ecological optimum in contaminated environments.

According to the ISA, RD communities showed highest similarity to RU communities and were composed by 53% of OTUs (88% of DNA reads) indicative of RU. However, the two river locations differed significantly. This can be explained by the continuous influence of micro- and macropollutants released by TEs that may inhibit the growth of sensitive species (Proia et al., 2013a), but also by possible species colonization downstream. Furthermore, 27% of the RD OTUs (9% of DNA reads) corresponded to indicator OTUs of U or H communities. Part of these OTUs probably corresponds to species that were released with the TEs and captured by the natural river biofilm downstream from the WWTP output. Motile, high-profile and especially planktic groups are more likely to disperse downstream with the water flow due to their lower resistance to current velocity (Liu et al., 2013; Dong et al., 2016). Indicator OTUs discriminating U or H and belonging to *Fragilaria*, *Gomphonema* (high-profile), and *Cyclotella* (planktic) were found in RD communities, despite their absence in RU. Transportation of free DNA from dead cells by the water flow may also occur and this DNA may be captured in river biofilms (e.g., Deiner et al., 2016; Pont et al., 2018). More directly, the permanent release of TEs causing constant pressure (PhC and nutrient load) in RD may favor the maintenance of more tolerant species and promote their stabilization in the biofilm.

River downstream communities seemed to be more influenced by U than by H communities, as 7% of the OTU reads found in RD corresponded to indicator OTUs of U, and only 2% – to indicator OTUs of H. The urban WWTP discharge was more than 15 times higher than the hospital one, which probably led to higher influence on water chemistry changes in RD.

#### Biological Diatom Index

Finally, molecular BDI was calculated from taxonomically assigned OTUs to evaluate the effect of TEs’ release on water quality in the recipient river. The application of BDI is usually limited to natural aquatic environments. However, WFD stations may be located in channels in urban environment build up with substrate different from natural river (similar to our case), and pollutant concentrations measured at TE sites during this study are comparable to these reported for highly polluted natural systems. Hence, we calculated BDI for all habitats (including TE sites) to evaluate the response of the entire diatom community to high anthropogenic pressure and track the effect of TEs on river communities. Compared to the river sites, communities in U and H reflected relatively low water quality (between “bad” and “moderate”), responding to the high anthropogenic pressure. WWTP discharges are generally monitored to avoid large alterations in river quality. The studied WWTP respected all national and European norms linked to removal efficiency and pollutants release (Chonova et al., 2016). Nevertheless, BDI based on molecular data suggested degradation of the water quality downstream from the WWTP output. This finding was confirmed by classical BDI based on microscopy counts (Sipibel Report, 2016) and it pointed out the efficiency of BDI to highlight changes in river ecology linked to the release of TEs.

## Conclusion

The present study shows that assessment of benthic diatoms using DNA metabarcoding is efficient to detect spatial and temporal community responses to pharmaceutical pressure in TEs of urban and hospital wastewaters and contributes to our understanding of the potential effluent impact on community dynamics when released in the recipient aquatic environment. We detected habitat-specific changes linked to the WWTP effluents on different community levels – richness, diversity, taxonomic composition, functional traits, and phylogenetic position. The changes in taxonomy and ecological traits included shift in proportion of polysaprobic motile groups in TE vs. oligosaprobic/oligotrophic groups belonging to the low- and high-profile guild in the river (especially upstream). RDA suggested that beta-blockers, NSAIDs, antibiotics and phosphate were among the most important factors driving community dynamics in TEs. ISA was helpful to evaluate the negative effect of these TEs on natural river community composition, revealing that 27% of OTUs detected in RD communities were indicative of urban or hospital treated effluent origin. Those OTUs may be either directly transferred with the TE and captured in the biofilm or maintained and developed further in RD due to the continuous release of TEs that changes chemical water parameters. Finally, the BDI calculated to evaluate the ecological status of the recipient river suggested that the release of TEs may lead to water quality decrease.

From these results, we can conclude that there is clearly a change in community structure linked to the WWTP effluents. As discussed above, studying environmental relationships between microbial communities and pollutants remains challenging due to occurring intercorrelations and the potential effect of confounding factors. Study on larger scale in combination with laboratory experiments would be helpful to explore and strengthen further relations between water chemistry and diatom community changes and to confirm findings reported here.

## Author Contributions

JL, AB, and TC contributed to the experimental design and sampling. TC contributed to the molecular experiments and data collection and organization. VV and TC contributed to the bioinformatics. RK, FK, FR, PI, and TC contributed to data analysis and statistics. RK, FR, JL, AB, and TC contributed to the interpretation of the data. FK and TC contributed to the preparation of the figures. RK, FR, JL, VV, FK, PI, AB, and TC contributed to the revision and approval of the manuscript.

## Conflict of Interest Statement

The authors declare that the research was conducted in the absence of any commercial or financial relationships that could be construed as a potential conflict of interest.

## Abbreviations

BDI: biological diatom index
COD: chemical oxygen demand
H: hospital treated effluent
HPLC–MS/MS: high performance liquid chromatography coupled to a mass spectrometer
HTS: high-throughput sequencing
ISA: indicator species analysis
NMDS: non-metric multidimensional scaling
NSAID: non-steroidal anti-inflammatory drug
OTU: operational taxonomic unit
PCR: polymerase chain reaction
PERMANOVA: permutational multivariate analysis of variance
PhC: pharmaceutical compounds
pRDA: partial redundancy analysis
RD: river downstream
RDA: redundancy analysis
RU: river upstream
TSS: total suspended solids
TEs: treated effluents
U: urban treated effluent
VIF: variance inflation factor
WFD: water framework directive
WWTP: wastewater treatment plant.
