# Supplementary material for: Benthic Diatom Communities in an Alpine River Impacted by Waste Water Treatment Effluents as Revealed Using DNA Metabarcoding
Source: Front Microbiol. 2019 Apr 9;10:653. doi: 10.3389/fmicb.2019.00653 (PMC6465766; doi:10.3389/fmicb.2019.00653)
Supplement: Supplementary file 1 [file Table_1.DOCX]

**Supplementary data**

**Table S1** – DNA sequencing data obtained from the sequencing platform after demultiplexing and adapter removal (standard deviation in brackets)

| Sample Name | Number of reads raw data | Number of reads after treatment | OTU number | OTU number after subsampling |
| --- | --- | --- | --- | --- |
| U-Feb | 53703 (6365) | 27150 (4781) | 140 (7) | 116 (2) |
| U-Mar | 62173 (5171) | 29726 (1854) | 144 (18) | 118 (13) |
| U-Apr | 59869 (1325) | 25552 (721) | 150 (7) | 121 (7) |
| U-May | 66410 (22429) | 33448 (18742) | 126 (13) | 106 (1) |
| U-June | 54235 (25082) | 18270 (6194) | 135 (28) | 122 (11) |
| U-July | 60943 (15554) | 23203 (10660) | 138 (14) | 123 (9) |
| H-Feb | 57394 (12702) | 17704 (2808) | 168 (4) | 152 (1) |
| H-Mar | 70030 (5802) | 23177 (1085) | 161 (10) | 138 (8) |
| H-Apr | 63668 (8921) | 18668 (3639) | 169 (21) | 153 (22) |
| H-May | 66978 (10839) | 20041 (409) | 168 (11) | 154 (9) |
| H-June | 68533 (10199) | 15680 (528) | 119 (6) | 110 (4) |
| H-July | 65061 (3285) | 14501 (894) | 136 (11) | 130 (11) |
| RU-Mar | 57963 (1442) | 24558 (2570) | 241 (15) | 203 (7) |
| RU-Apr | 50011 (2660) | 22271 (1698) | 238 (6) | 202 (5) |
| RU-May | 40912 (5991) | 16317 (1841) | 269 (29) | 250 (20) |
| RU-June | 63518 (5004) | 23181 (2943) | 308 (33) | 270 (28) |
| RU-July | 50715 (10276) | 17282 (3976) | 277 (43) | 262 (29) |
| RD-Mar | 59428 (2844) | 23471 (1710) | 220 (37) | 187 (28) |
| RD-Apr | 53822 (6087) | 19823 (2966) | 285 (23) | 260 (17) |
| RD-May | 66610 (4135) | 20669 (2436) | 201 (80) | 183 (74) |
| RD-June | 61245 (6571) | 21669 (4095) | 260 (40) | 237 (34) |
| RD-July | 59610 (3850) | 22208 (3001) | 224 (23) | 203 (22) |
